# Calcareous Concretions in the External Ear

**Published:** 1887-05

**Authors:** Lester Curtis

**Affiliations:** 35 University Place, Chicago


### DOMESTIC CORRESPONDENCE.

### CALCAREOUS CONCRETIONS IN THE EXTERNAL EAR.

*To the Editor of the Chicago Medical Journal and Examiner.*

The following case, occurring in an old patient of mine, has seemed to me to be unusual. I had treated the patient for several years for slight ailments, chief of which was an occasional malarial attack that gave me some trouble on account of her inability to take quinine.

Last winter she suffered from the vomiting of pregnancy. I treated the case for about two months without result, when, as the symptoms became alarming, Dr. Byford, at my request, brought on a miscarriage. She was, of course, much prostrated and her stomach remained for a long time irritable and there were distinct evidences of spinal anæmia, neither of which symptoms have yet entirely disappeared.

Her recovery, though slow, was uninterrupted, with the exception of an induration on the calf of the left leg where a hypodermic needle had been inserted. This induration gradually assumed the size of half a pullet's egg, became red and tender, and finally fluctuated distinctly under the finger. I delayed opening it, and, under the application of poultices it finally disappeared, leaving a depression somewhat more than an inch across. The depression still remains. I give these details, as they may have some bearing on the subsequent history of the case.

In August last, she called my attention to a painful swelling in the external meatus of the right ear. I considered it to be a case of ordinary superficial inflammation of the meatus, and directed instillation of a solution of morphine. The pain at once ceased, and in a day or two the swelling subsided and I supposed that the trouble had resolved, as has usually occurred in my experience, after such treatment. But a little swelling and tenderness remained. In about two weeks, without further symptoms than a trifling uneasiness, there was a discharge of a drop of purulent fluid which I am sorry that I had no opportunity of examining. I paid no more attention to the case until about a month after this, when she called at my office and told me that the same morning the ear had felt a little uncomfortable, and on picking it a calcareous mass about as large as a millet seed had come away. A day or two before she had removed a similar mass. These masses, she said, resembled lumps of old lime as nearly as anything she could think of.

On examining the ear I saw a red and indurated place about half way down the meatus in the upper portion of the posterior wall. In the middle of this spot was a pit about as large as the mass removed, and surrounding the pit a greater or less amount of a white granular substance, which grated against the probe and was of stony hardness. I attempted to detach some of it with the sharpened end of the probe, but it was firmly adherent, and appeared to be infiltrated throughout the tissue, and I succeeded in getting away only a few small flakes. Besides this, nothing unusual was detected in the ear with the exception of a slight redness of the posterior rim of the drum. The hearing was perfect. A slight feeling of irritation was noticed for some weeks, but gradually passed away. At present, so far as I can discover, the ear is quite normal.

I do not think that there is any possibility of the concretion being a foreign body; nothing with the exception of the morphine was introduced into the ear, and that could hardly have produced such an effect; besides, part at least of the substance was infiltrated among the tissues of the meatus.

Superficial abscesses where there is a free discharge of pus do not undergo calcareous degeneration.

The appearance was that of a gouty concretion, and there is a trace of gout in the family, her father,—though an active and abstemious man—having had slight attacks of the affection ; but she has never had any gouty symptom that I have been able to discover, and gout does not suppurate.

I have never heard of a case like this, and the somewhat limited amount of literature on diseases of the ear accessible to me makes no mention of anything of the kind.

LESTER CURTIS, M.D.

35 University Place, Chicago.

